# The effect of age on the clinical characteristics and innate immune cell function in the patients with abdominal sepsis

**DOI:** 10.3389/fphys.2022.952434

**Published:** 2022-09-27

**Authors:** Jiaqi Lu, Jingyuan Liu, Liuluan Zhu, Yue Zhang, Ang Li

**Affiliations:** ^1^ Department of Critical Care Medicine, Beijing Ditan Hospital, Capital Medical University, Beijing, China; ^2^ Beijing Key Laboratory of Emerging Infectious Diseases, Institute of Infectious Diseases, Beijing Ditan Hospital, Capital Medical University, Beijing, China; ^3^ Beijing Institute of Infectious Diseases, Beijing Ditan Hospital, Capital Medical University, Beijing, China; ^4^ National Center of Integrative Medicine, Beijing Ditan Hospital, Capital Medical University, Beijing, China

**Keywords:** sepsis, abdominal infection, age, clinical features, innate immunity

## Abstract

Sepsis is a life-threatening dysregulated host response to infection that compromises organ health, and abdominal sepsis is a commonly presenting critical illness in intensive care units (ICU). In this study, we investigate the effect of age on clinical sepsis characteristics and innate immune cells (neutrophils and monocytes) functionality in abdominal sepsis patients. We recruited 32 patients with abdominal sepsis from the Beijing Ditan Hospital’s ICU from February 2021 to September 2021, and selected 18 healthy volunteers that were age- and sex-matched as controls for a prospective cohort study. Elderly abdominal sepsis patients (age >65 years) had the following altered characteristics compared to nonelderly patient controls: lower mean arterial pressure, monocytes percentage, and red blood cell volume distribution width (*p* < 0.05); higher neutrophils percentage and neutrophils-to-lymphocytes ratio (*p* < 0.05); significantly increased monocyte-produced reactive oxygen (*p* < 0.05); increases neutrophilic secretion of TNF-α, as well as lower monocytic secretion of TNF-α (*p* < 0.05); higher neutrophil percentage (which was significantly higher in peripheral blood than monocyte percentage). Elderly patients also had significantly increased phagocytic activity in their neutrophils and monocytes (*p* < 0.05), significantly reduced neutrophils-produced reactive oxygen (*p* < 0.001), and significantly increased TNF-α secretion by monocytes and neutrophils (*p* < 0.05). We found that elderly patients have decreased immune cell function and increased release of cytokines compared to younger patients, suggesting individualized treatment plans targeting the elderly septic microenvironment could help prevent organ failure in elderly septic patients and improves patient survival.

## Introduction

As people age, their ability to host a dynamic and effective immune response is dampened, increasing the odds of developing opportunistic and pathogenic bacterial infections associated with poor outcomes (Girard et al., 2005). Treatment of infection in elderly patients is often delayed due to their atypical symptom presentation, further worsening their prognosis. In the United States, the elderly population (65 + years of age) accounts for 58%–65% of sepsis cases (Dombrovskiy et al., 2007). Over time, the elderly population has steadily increased and the medical expenditure associated with elderly sepsis treatment follows a similar trend (Starr and Saito, 2014). Elderly patients are also more likely to experience early death due to various other factors, such as underlying organ dysfunction and compromised immunity, and even survivors require more clinical care and long-term organ function support. Elderly sepsis patients have a high rate of complications and poor long-term survival rate compared to younger patients (Carbajal-Guerrero et al., 2014). Intra-abdominal infection (IAI) is a common disease in the intensive care units (ICU), which can occur secondary to cirrhosis, gastrointestinal perforation, necrosis and gangrene, suppurative appendicitis, appendiceal perforation, intestinal obstruction, and intestinal necrosis. Severe IAI can easily trigger a systemic inflammatory response due to the entry of a large number of bacteria over a short period of time (Mazuski et al., 2017). IAI is a major contributing factor to the development of sepsis in ICU patients and is also associated with rapid disease progression. Patient outcomes can be improved by noticing important IAI-related factors in sepsis, avoid the increased possibility of sepsis, and finally improve the quality of life of patients with IAI-related sepsis in ICU.

As the immune system ages it begins to undergo immunosenescence. This causes a variety of physiological, biochemical, and hormonal changes that cause an imbalance within the immune system. This imbalance is responsible for the increased susceptibility of elderly patients to infection, reduced efficacy of vaccination, and a chronic low-level inflammatory state (Fülöp et al., 2016; Sadighi Akha, 2018). Elderly patients with additional pre-existing conditions, such as diabetes, malignant tumors, heart, kidney, or liver diseases, are more likely to develop sepsis once abdominal infection occurs (Chen et al., 2018). The immunosuppressive state induced by intra-abdominal sepsis is confirmed in a growing number of experimental studies (Vincent et al., 2020) and in clinical case studies on sepsis patients. Neutrophils and monocytes are key effector cells of the innate immune response and play an important role in host defense. These cells can recognize and phagocytose pathogens, produce inflammatory mediators, and initiate other immunological mechanisms, such as the recruitment of polymorphonuclear cells from peripheral blood to the site of infection, that ultimately lead to resolution of infection. The main objective of this prospective study was to investigate the effect of age on clinical sepsis characteristics as well as neutrophil and monocyte function in abdominal sepsis patients. Accurate understanding of changes in clinical characteristics and immune function in elderly patients with abdominal sepsis can help improve prognosis by facilitating timely diagnosis and selection of the best treatment.

## Materials and methods

### Study subjects and grouping

In this study, we included 32 abdominal infection patients with sepsis that were recruited from the Beijing Ditan Hospital ICU between February 2021 and September 2021. These patients were divided into two groups, the elderly group (≥65 years, 14 patients) and the non-elderly group (<65 years, 18 patients), based on the reported age threshold (Narci et al., 2009) for epidemiological analysis. We compared the neutrophil and monocyte phagocytic function, neutrophil and monocyte respiratory burst function, neutrophil and monocyte tumor necrosis factor- alpha (TNF-α) release function, and neutrophil and monocyte phenotypes between patients and healthy controls (*n* = 18). We also compared the clinical features, neutrophil and monocyte functions, phenotype, and outcome measures of patients on the first day of diagnosis with abdominal sepsis between the elderly group and the non-elderly group. Patients were tracked until December 2021 to record their infectious events, the development of organ failure, 90-day survival, hospitalization, and ICU stay.

### Inclusion criteria

In this study we included patients between the ages of 18 and 80 who were diagnosed with abdominal sepsis during hospitalization. Diagnostic criteria for abdominal sepsis were: 1) deemed septic by the Sequential Organ Failure Assessment (SOFA) score where sepsis is defined by a score increase of 2 or equal to 2. For screening purposes, rapid SOFA scores based on three criteria for neurologic (Glasgow Coma Scale <15), cardiovascular (systolic blood pressure <100 mmHg), and respiratory (respiratory rate >22/min) dysfunction are available clinically identified patients at high risk of sepsis. The diagnostic criteria for septic shock were that vasopressor drug therapy is initiationly and continuously needed to maintain a mean arterial pressure >65 mmHg or a serum lactate level >2 mmol/L, and that the infection site is in the abdominal cavity. 2) There are at least two ways to diagnose abdominal infection: such as history taking, physical examination, laboratory examination, imaging examination and abdominal paracentesis. History taking and physical examination can help diagnose patients with suspected IAI, and laboratory tests can further identify the degree of disease progression. Among them, symptoms and signs such as abdominal pain, rebound pain, cessation of flatus and defecation by anus, and fever all have a strong suggestive effect on abdominal infection, while white blood cell (WBC) count, C-reactive protein (CRP), and procalcitonin (PCT) can predict the degree of infection progression. Imaging examination and paracentesis is important for the localization of the source of infection, the severity of the lesion, and the selection of subsequent treatment measures. 3) Healthy, age- and sex-matched, non-smoking volunteers with no history of liver disease served as healthy controls (HC). HC alcohol intake was <20 g/day and volunteers did not drink alcohol or exercise excessively within 24 h before blood sampling. 4) The above tests were carried out with the informed consent of the patients and their families and were approved by the Medical Ethics Committee.

### Exclusion criteria

The following patients were excluded from this study: 1) patients with incomplete clinical data; 2) patients who took antibiotics for at least 5 days due to suspected infection in the past 30 days; 3) patients with a history of any immunodeficiency including malignancy or human immunodeficiency virus (HIV); 4) patients treated with immunosuppressive agents (prednisolone, azathioprine); 5) patients with successful resuscitation after sudden cardiac arrest; and 6) pregnant women.

### Ethics, informed consent, and data collection

This study was approved by Committee of Ethics at Beijing Ditan Hospital, Capital Medical University [Department of medical ethics; Beijing Ditan Hospital (2021)-005]. Blood samples, clinical, biochemical data, and physiological data of patients were collected after obtaining fully informed consent. The blood samples of the HC group were from the Health Examination Center of Beijing Ditan Hospital.

### Flow cytometry

We collected 4 ml of peripheral blood from sepsis patients and HC, added it into Ethylene Diamine Tetraacetic Acid (EDTA) anticoagulant tubes and immediately pre-cooled samples to 0°C–4°C. We performed the neutrophil and monocyte function analysis within 2 h of blood being drawn. The solution was aspirated into a centrifuge tube and the erythrocytes were lysed with ammonium chloride lysis buffer (BioLegend, United States) and washed with phosphate buffered saline (PBS) (Corning, United States). Single cells were obtained by adding 10% fetal bovine serum (Gibco, United States) and Roswell Park Memorial Institute (RPMI) 1640 medium containing 1% penicillin and streptomycin (Corning, United States) for *in vitro* culture and incubated in a carbon dioxide incubator for 30 min. Flow cytometry was performed on cells with the following antibodies: PE anti-CD16 (BioLegend), APC anti-CD14 (BioLegend, United States), PerCP anti-CD45 (BioLegend, United States), FITC anti-CD16 (BioLegend, United States), BV421 anti-TNF-α (BioLegend, United States). Latex Beads (Carboxylate-modified, Yellow-green, Sigma) were used for cellular phagocytic function analysis. The respiratory burst of cells was detected with 2,7-dichlorofluorescein diacetate (DCFH-DA. Sigma). Samples were processed through a BD FACSCanto flow cytometer and analyzed. After obtaining 50,000–100,000 cells per sample, cells were selectively gated by forward scattering versus side scatter dot plots. Flowjo V10 software was used to analyze the flow cytometry data, and GraphPad Prism7 was used to perform the graphing and statistical analysis.

### Phagocytic test

Yellow-green-labeled Latex beads were added to the peripheral blood single cell suspension, incubated in a carbon dioxide incubator at 37°C for 3 h, placed on ice for 10 min to stop phagocytosis, and then washed twice with PBS buffer. Next, the corresponding cell surface fluorescent antibody combination was added (CD16-PE/CD14-APC/CD45-PerCP staining) and allowed to incubate at room temperature for 15 min in the dark. PBS buffer was added after the incubation period for washing. The ratio of Beads+ cells in neutrophils and monocytes was detected and analyzed by flow cytometer to determine the phagocytic function.

### Determination of respiratory burst

Peripheral blood single-cell suspensions from patients and age-matched healthy controls were simultaneously assayed with 0.5 mg/ml DCFH-DA. The peripheral blood single cell suspension was incubated in a carbon dioxide incubator at 37°C for 15 min, fixed with 4% paraformaldehyde for 10 min, washed twice with PBS buffer, and then received a combination of neutrophil fluorescent antibodies (CD16-PE/CD14-APC/CD45-PerCP staining), Samples were incubated at room temperature for 15 min in the dark, washed with PBS buffer, and then run through the flow cytometer to analyze the proportion of DCFH+ cells in neutrophils and monocytes, and measure the generation of reactive oxygen species (ROS) in that percentage of cells.

### Determination of intracellular factor tumor necrosis factor- alpha

Golgi plug (Thermal Fisher, United States) was added to the peripheral blood single-cell suspension to block the secretion of cytokines. Golgi plug was incubated with cells at 37°C in a carbon dioxide incubator for 3 h, and the neutrophil fluorescent antibody combination (CD16-FITC/CD14-APC/CD45-PerCP staining) was added, incubated at room temperature for 15 min in the dark, and then washed with PBS buffer. Next, we added the intracellular fluorescent staining kit Fix-Perm (BD bioscience, United States) membrane breaking agent, incubated at 4°C for 20 min in the dark, used Perm Wash (Becton Dickinson, United States) to rinse twice, added intracellular protein fluorescent antibody TNF-α-BV421, incubated at 4°C in the dark for 30 min, and, lastly, washed twice with Perm Wash. We used flow cytometry to analyze the ratio of TNF-α+ in neutrophils and monocytes, and measure the function of producing TNF-α.

### Statistical analysis

The data of subjects with normal distribution were expressed in the form of mean ± standard deviation. The difference in unpaired data between two groups was analyzed by independent sample *t*-test, and the paired data was analyzed by paired sample *t*-test, Data for more than two groups were analyzed by one-way ANOVA. Data that was not normally distributed was expressed by median and interquartile range. The unpaired data between two groups was analyzed by Mann-Whitney test, and the paired data was analyzed by Wilcoxon matched-pairs signed rank test. For all data, *p* < 0.05 was considered statistically significant.

## Results

### Baseline demographic characteristics and clinical parameters of patients in different age groups

Among the 32 recruited patients, 14 (43.8%) were elderly and 18 (56.2%) were non-elderly, with no significant difference in gender between these two groups (*p* = 0.458). As shown in Table1, 64.3% (9/14) of the elderly patients but only 44.4% (8/18) of the non-elderly patients had underlying diseases (*p* = 0.308). Septic shock was more common in the elderly group (78.6%) than in the non-elderly group (50%), but no significant difference was detected for this trend (*p* = 0.098). By comparing the basic vital signs of patients on the first day of diagnosis, it was found that the mean arterial pressure distribution of the elderly group and the non-elderly group was 60 (56–70) mmHg and 73 (64–81) mmHg, respectively, which was significantly different (*p* = 0.020). In addition, the comparison of routine blood parameters between the two groups demonstrated that the percentage of neutrophils in the elderly group on the first day [88.0% (83.1%–91.7%)] were significantly higher than those in the non-elderly group [83.7% (73.3%)]-87.1%)] (*p* = 0.017). The monocytes percentage [5.3% (3.7%–7.1%)], however, was significantly lower in the elderly group when compared to the non-elderly group [8.3% (5.0%–13.2%)] (*p* = 0.040). The neutrophil to lymphocyte ratio (NLR), derived from complete blood cell count, has been shown to be a widely used non-specific inflammatory marker, and recent studies have found that the addition of platelet count to this ratio, (the neutrophil to lymphocyte and platelet ratio, NLPR), is expected to further improve its clinical value. In this study, our results showed that the NLRs of elderly patients [14.07 (9.48–27.40)] were significantly higher than those of non-elderly patients [11.38 (5.08–15.18)] (*p* = 0.003). Meanwhile, the NLPRs of patients in the elderly group [14.59 (8.70–24.95)] were also higher than those of patients in the non-elderly group [10.59 (7.16–16.66)], but the difference was not statistically significant (*p* = 0.067). In addition, the red blood cell volume distribution widths in the elderly group [15.4% (13.1%–17.6%)] were significantly lower than those in the non-elderly group [18.2% (14.3%–22.3%) (*p* = 0.044)].

**TABLE 1:** Basic clinical characteristics of patients with abdominal sepsis in different age groups.

		Total (*n* = 32)	Group A (≥65; *n* = 14)	Group B (<65; *n* = 18)	*p*
Demographic characteristics	Male (%)	21 (65.6)	9 (64.2)	12 (66.7)	0.458
Basic disease	Basic diseases (%)	17 (53.1)	9 (64.3)	8 (44.4)	0.308
	Hypertension (%)	13 (40.6)	9 (64.3)	4 (22.2)	0.149
	Diabetes (%)	7 (21.9)	4 (28.6)	3 (16.7)	0.669
	CHD (%)	4 (12.5)	3 (21.4)	1 (5.6)	0.295
	CRF(%)	6 (18.8)	4 (28.6)	2 (11.1)	0.365
Operation	Operation (%)	4 (12.5)	2 (14.3)	2 (11.1)	0.788
Septic shock	Shock (%)	20 (62.5)	11 (78.6)	9 (50.0)	0.098
SOFA score	SOFA	6 (5–8)	6 (4–8)	6 (5–9)	0.283
Basic vital signs	Temperature (°C)	37.5 ± 0.8	37.3 ± 0.8	37.7 ± 0.7	0.106
	HR (per minute)	89 ± 17	85 ± 17	93 ± 16	0.204
	MAP (mmHg)	67 (60–77)	60 (56–70)	73 (64–81)	0.020
	RR (per minute)	17 (16–20)	18 (17–21)	17 (16–19)	0.210
	SPO_2_(%)	97 (95–99)	97 (95–98)	98 (96–99)	0.465
Internal environment	PH	7.45 ± 0.08	7.44 ± 0.08	7.47 ± 0.08	0.287
	PCO_2_(mmHg)	35 (28–38)	36 (29–41)	34 (28–37)	0.262
	PO_2_(mmHg)	105 ± 26	106 ± 29	104 ± 24	0.239
	BE (mmol/L)	0.8 ± 5.2	0.6 ± 4.5	1.0 ± 5.9	0.852
	HCO3^-^(mmol/L)	24.5 ± 4.8	24.5 ± 4.1	24.5 ± 5.5	0.998
	Lactate (mmol/L)	1.5 (1.0–2.2)	1.4 (1.0–2.1)	1.6 (1.1–2.2)	0.676
Blood routine index	WBC(10^9^/L)	10.4 (6.8–16.7)	11.1 (7.6–14.6)	10.1 (6.7–18.2)	0.761
	NE (%)	85.7 (78.7–89.7)	88.0 (83.1–91.7)	83.7 (73.3–87.1)	0.017
	NEUT (10^9^/L)	8.7 (5.4–14.2)	9.1 (6.9–13.0)	7.6 (5.0–15.6)	0.470
	LY (%)	7.2 (5.6–9.2)	6.2 (3.4–8.8)	7.5 (5.7–13.8)	0.091
	LYM(10^9^/L)	0.8 (0.4–1.2)	0.7 (0.3–1.0)	0.9 (0.4–1.7)	0.086
	MO(%)	6.2 (4.4–10.2)	5.3 (3.7–7.1)	8.3 (5.0–13.2)	0.040
	MONO(10^9^/L)	0.7 (0.3–1.1)	0.5 (0.3–1.0)	0.9 (0.3–1.5)	0.231
	NLR	12.24 (8.73–15.65)	14.07 (9.48–27.40)	11.38 (5.08–15.18)	0.003
	NLPR	12.65 (7.54–21.07)	14.59 (8.70–24.95)	10.59 (7.16–16.66)	0.067
	Hemoglobin (g/L)	92 ± 27	94 ± 23	92 ± 31	0.836
	HCT (%)	27.4 ± 7.7	27.7 ± 6.5	27.1 ± 8.6	0.837
	MCV(fl)	90 ± 9	93 ± 7	88 ± 10	0.177
	MCH(pg)	30.7 (28.9–32.8)	30.9 (29.2–32.4)	30.0 (27.9–33.0)	0.403
	MCHC(g/l)	337 ± 17	338 ± 12	336 ± 21	0.845
	RDW (%)	16.8 (13.7–20.3)	15.4 (13.1–17.6)	18.2 (14.3–22.3)	0.044

Note: Group A indicates the cohort of patients aged ≥65 years. Group B indicates the cohort of patients aged <65 years. CHD, coronary heart disease; CRF, chronic renal failure; SOFA, sequential organ failure assessment; HR, heart rate; MAP, mean Arterial pressure; RR, respiratory rate; SPO_2_, Arterial oxygen saturation; PH, potential of hydrogen; PCO_2_, partial pressure of carbon dioxide; PO_2_, oxygen partial pressure; BE, base excess; HCO_3_^−^, bicarbonate radical; WBC, white blood cell count; NE, neutrophil percentage; NEUT, neutrophil count; LY, percentage of lymphocytes; LYM, lymphocyte count; MO, monocyte percentage; MONO, monocyte count; NLR, neutrophil to lymphocyte ratio; NLPR, neutrophil to lymphocyte and platelet ratio; HCT, hematokrit; MCV, mean corpuscular volume; MCH, mean hemoglobin; MCHC, mean hemoglobin concentration; RDW, red blood cell distribution width.

The comparison of organ function indicators on the first day of diagnosis of sepsis in the two groups showed that the levels of total bilirubin (liver function indicator) in the non-elderly group were higher than those in the elderly group, which were 60.7 (21.0–117.2) mmol/L and 15.5 (9.0–32.3) mmol/L, respectively (*p* = 0.015). However, the differences in renal function, coagulation function, cardiac function, acid-base electrolyte, and some infection indexes were not found to be statistically significant (*p* > 0.05) (Table 2).

**TABLE 2:** Visceral function measures in abdominal sepsis patients of different age groups.

		Total (*n* = 32)	Group a (≥65; *n* = 14)	Group B (<65; *n* = 18)	*p *
Liver function index	TBIL (umol/L)	27.1 (14.0–92.7)	15.5 (9.0–32.3)	60.7 (21.0–117.2)	0.015
	DBIL (umol/L)	15.4 (7.0–68.7)	8.0 (6.0–17.1)	32.9 (13.3–100.1)	0.019
	Albumin (g/L)	33.0 ± 4.9	34.5 ± 3.5	31.9 ± 5.6	0.141
	Cholinesterase (U/L)	2441 (1553–3989)	2648 (1864–4075)	2364 (1146–3749)	0.425
	ALT (U/L)	34.7 (11.0–71.7)	22.5 (7.4–68.1)	38.0 (11.9–108.2)	0.333
	AST (U/L)	41.8 (18.6–87.3)	39.3 (16.3–68.1)	43.8 (20.5–117.1)	0.543
	LDH(U/L)	233.8 (176.6–350.0)	233.8 (168.3–304.1)	222.4 (159.4–438.5)	0.739
	CK(U/L)	59.5 (29.3–338.2)	95.2 (36.2–749.2)	58.0 (22.9–260.1)	0.257
Coagulation function index	PLT (10^9^/L)	105 (66–158)	140 (75–185)	97 (58–135)	0.119
	PT(s)	16.5 (13.2–22.5)	15.1 (13–21.2)	18.2 (13.7–23.1)	0.436
	PTA (%)	55 ± 21	58 ± 23	52 ± 19	0.478
	APTT(s)	36.2 (29.2–42.3)	33.3 (28.7–42.6)	36.4 (31.8–43.7)	0.403
	Fib (mg/dl)	279 (150–420)	335 (163–457)	193 (132–338)	0.087
	INR (%)	1.5 (1.2–2.0)	1.4 (1.2–2.0)	1.7 (1.3–2.0)	0.436
	FDP (ug/mL)	11.8 (7.1–24.1)	11.4 (8.1–16.7)	13.3 (4.9–49.1)	0.820
	D - dimer (mg/L)	3.8 (1.9–11.5)	3.8 (2.8–6.8)	4.3 (1.6–18.0)	0.924
	PT(s)	17.6 (14.5–19.6)	17.1 (14.2–19.3)	18.0 (14.6–19.7)	0.648
Renal function index	K^+^(mmol/L)	3.8 ± 0.5	4.0 ± 0.5	141.5 ± 7.0	0.097
	Na^+^(mmol/L)	104.2 ± 6.1	138.7 ± 4.3	27.1 ± 8.6	0.207
	CL^−^(mmol/L)	103.5 (100.6–107.9)	102.5 (99.7–105.8)	104.7 (101.3–113.3)	0.210
	Ca^2+^(mmol/L)	2.1 ± 0.2	2.1 ± 0.2	2.1 ± 0.2	0.776
	Mg^2+^(mmol/L)	1.0 ± 0.2	1.0 ± 0.2	0.9 ± 0.2	0.087
	Carbamide (mmol/L)	9.8 (6.4–17.2)	10.0 (5.9–14.4)	9.8 (6.5–22.4)	0.470
	Creatinine (umol/L)	107.0 (69.9–172.6)	120.7 (62.0–182.8)	107.0 (70.3–171.1)	0.790
	Blood Glucose (mmol/L)	10.5 ± 3.2	11.4 ± 3.2	9.7 ± 3.2	0.148
	AG (mmol/L)	16.6 ± 4.1	16.6 ± 3.4	16.6 ± 4.6	0.988
	Crystalloid Osmotic Pressure	301.9 (294.1–310.1)	300.3 (295.1–307.8)	304.6 (293.2–314.3)	0.382
	GFR (ml/min/1.73 m2)	56.7 (30.9–98.4)	51.3 (28.4–94.5)	56.7 (36.8–102.6)	0.518
Cardiac function index	BNP(pg/ml)	478.3 (65.3–770.9)	250.8 (39.5–1104.0)	481.7 (81.2–654.8)	0.896
	Myohemoglobin (ng/ml)	126.7 (89.3–368.4)	170.3 (88.1–373.3)	122.6 (96.3–340.1)	0.868
	TNI(ng/mL)	0.038 (0.012–0.195)	0.023 (0.012–0.076)	0.054 (0.011–12.059)	0.984
	CK-MB(ng/mL)	2.1 (0.6–8.1)	2.4 (0.9–8.1)	1.9 (0.5–7.5)	0.510
Infection index	PCT (ng/mL)	1.8 (0.5–6.2)	1.3 (0.6–4.4)	2.5 (0.4–6.5)	0.494
	CRP (mg/L)	68.0 (34.1–115.6)	82 (40.1–118.3)	49.1 (15.6–108.2)	0.153

Note: Group A indicates the cohort of patients aged ≥65 years. Group B indicates the cohort of patients aged <65 years. TBIL, total bilirubin; DBIL: direct bilirubin; ALT, alanine transaminase; AST, aspartate Aminotransferase; LDH, lactic dehydrogenase; CK, creatine kinase; PLT, platelet count; PT, prothrombin time; PTA, prothrombin activity; APTT, activated partial thromboplastin time; Fib, Fibrinogen; INR, international normalized ratio; FDP, fibrinogen degradation product; K^+^, serum kalium; Na^+^, Natriumion; CL^−^, chloridion; Ca^2+^, Calciumion; Mg^2+^, Magnesiumion; AG, anion gap; GFR, glomerular filtration rate; BNP, B Type Natriuretic Peptide; TNI, Hypersensitive Troponin I; CK-MB, Creative kinase Isoenzyme MB; PCT, procalcitonin; CRP, C-reactive protein.

### Phagocytic function of innate immune cells

**FIGURE 1:**
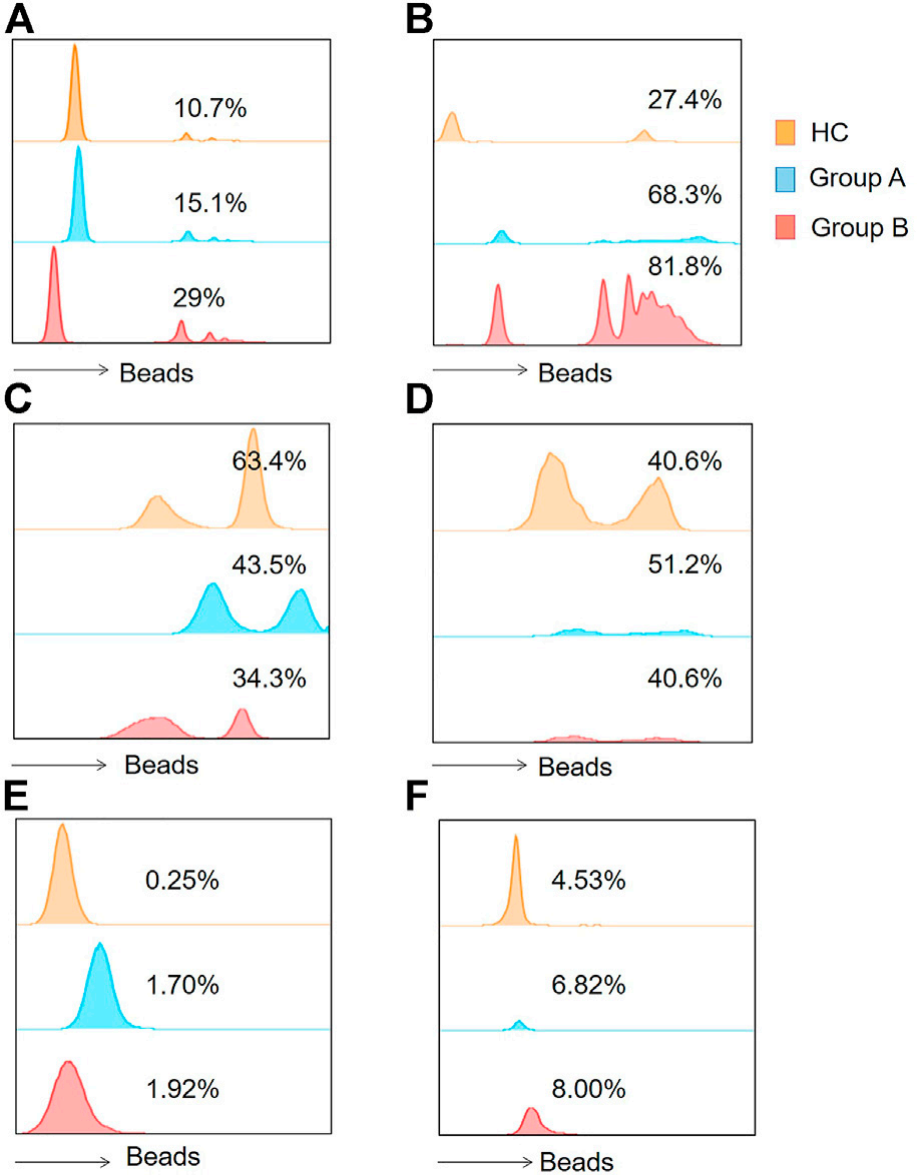
Histograms of flow cytometry were applied to represent the functions of the two immune cells. **(A)** Indicates the phagocytic function of neutrophils. **(B)** Indicates monocyte phagocytic function. **(C)** Represents the neutrophil ROS production results. **(D)** Represents monocyte ROS production results. **(E)** Represents neutrophil intracellular TNF- α production proportions. **(F)** Indicates monocytic intracellular TNF- α production proportions. Group A indicates the cohort of patients aged ≥65 years. Group A indicates the cohort of patients aged <65 years. Tumor Necrosis Factor- alpha, TNF-α; Reactive Oxygen Species, ROS.

**FIGURE 2:**
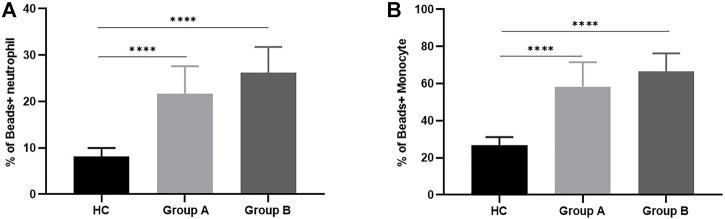
Phagocytosis of neutrophils and monocytes in healthy controls and patients with abdominal sepsis of different age groups. Note: **(A)** Represents the phagocytic function of neutrophils. **(B)** Represents the phagocytic function of monocytes. Group A indicates the cohort of patients aged ≥65 years. Group A indicates the cohort of patients aged <65 years. We utilize how many of the asterisks indicate the magnitude of the *p*-value. “****” indicates *p* < 0.0001.

Neutrophils and monocytes are important components of the innate immune response in sepsis, both of which have phagocytic function. Once phagocytes find and recognize pathogens, phagocytosis will occur, and then phagocytic enzyme activity increases. In order to understand the phagocytic function of patients with abdominal sepsis in different age groups, the phagocytic function of phagocytes collected on the first day of diagnosis of abdominal sepsis was detected by flow cytometry (Figures 1A,B), and compared with that of healthy volunteers (Figure 2). We found that the phagocytic function of both neutrophils and monocytes was elevated in patients with peritoneal sepsis compared to healthy volunteers (*p* < 0.001). In terms of neutrophils (Figure 2A), the phagocytic function of elderly abdominal sepsis patients was lower than that of non-elderly patients (*p* > 0.05). Similarly, the phagocytic function of monocytes in the elderly group was lower than that of the non-elderly group (*p* > 0.05) (Figure 2B).

### Respiratory burst of innate immune cells

**FIGURE 3:**
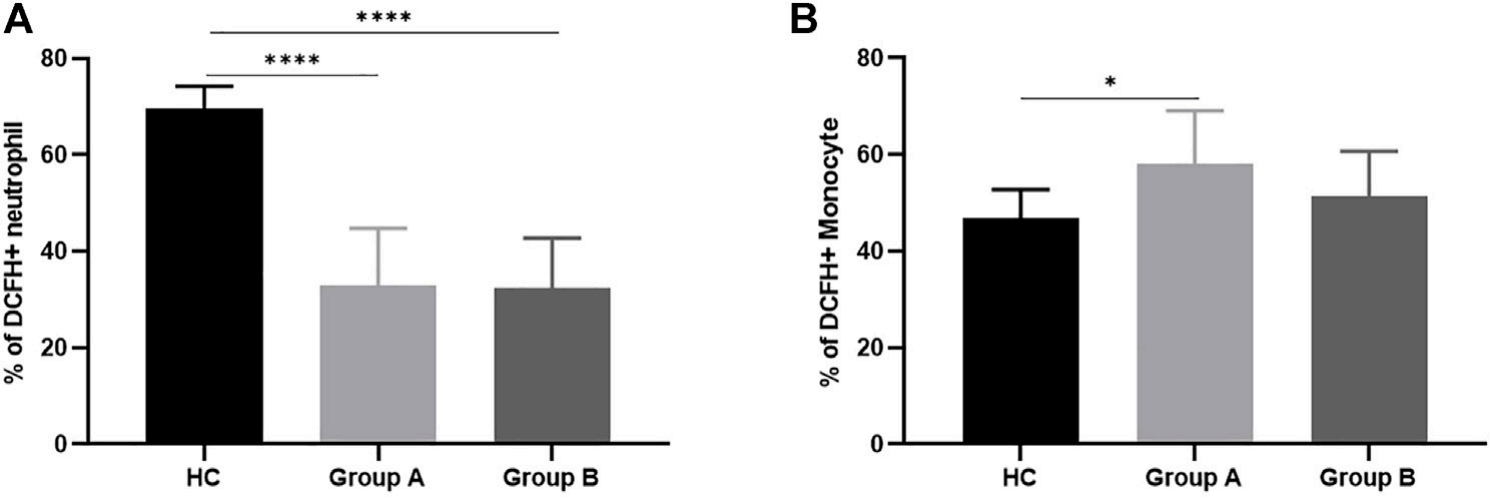
ROS production by neutrophils and monocytes in healthy controls and patients with abdominal sepsis of different age groups. Note: **(A)** Represents ROS of neutrophils. **(B)** Represents ROS of monocytes. Group A indicates the cohort of patients aged ≥65 years. Group A indicates the cohort of patients aged <65 years. Reactive Oxygen Species, ROS. We utilize how many of the asterisks indicate the magnitude of the *p*-value. “*” indicates *p* < 0.05. “****” indicates *p* < 0.0001.

**FIGURE 4:**
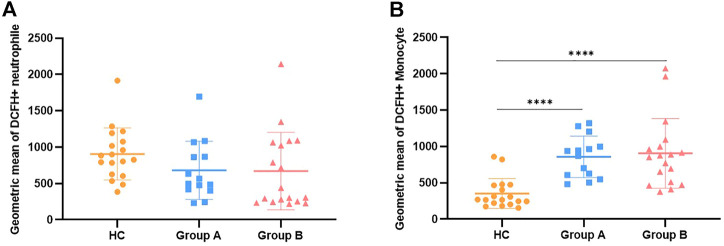
Description of ROS generation results by mean fluorescence intensity along with comparison. Note: **(A)** Represents the mean fluorescence intensity of the results of ROS production by neutrophils. **(B)** Represents the mean fluorescence intensity of the results for ROS production by monocytes. Group A indicates the cohort of patients aged ≥65 years. Group A indicates the cohort of patients aged <65 years. Reactive Oxygen Species, ROS. We utilize how many of the asterisks indicate the magnitude of the *p*-value. “****” indicates *p* < 0.0001.

In order to clarify the changes of respiratory burst function of phagocytes and further explore their actual ability to eliminate pathogenic microorganisms, we detected the production of cellular ROS (Figures 1C,D). The production of ROS by neutrophils was significantly reduced in both elderly and non-elderly patients with abdominal sepsis compared with HC group (Figure 3A) (*p* < 0.001), but the decrease degree between the elderly group and non-elderly group was not statistically significant (*p* > 0.05). As seen from Figure 3B, the monocyte ROS production in elderly patients was significantly higher than that of the non-elderly group (*p* < 0.05), and the production of ROS by monocytes in the non-elderly group was higher than healthy volunteers, however the difference was not statistically significant (*p* > 0.05). In addition, we employed the mean fluorescence intensity to profile the results of cellular ROS production (Figure 4).

### Intracellular tumor necrosis factor- alpha release from innate immune cells

**FIGURE 5:**
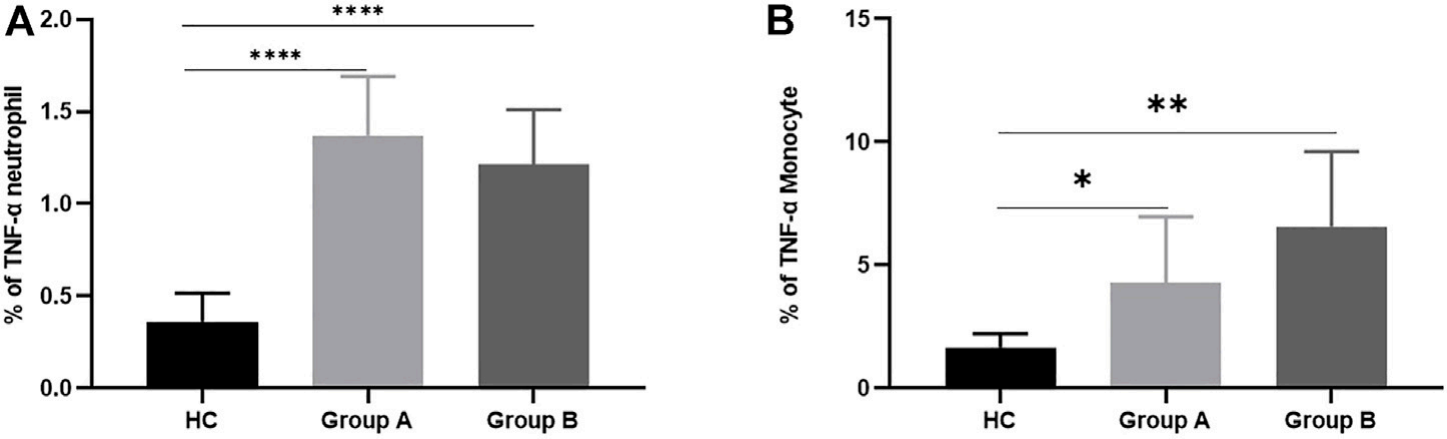
TNF-α production from neutrophils and monocytes in healthy controls and patients with abdominal sepsis of different age groups. Note: **(A)** represents TNF- α secretory **function of** neutrophils. **(B)** represents TNF- α secretory **function of** monocytes. Group A indicates the cohort of patients aged ≥65 years. Group A indicates the cohort of patients aged <65 years. Tumor Necrosis Factor- alpha, TNF-α. We utilize how many of the asterisks indicate the magnitude of the *p*-value. “*” indicates *p* < 0.05. “**” indicates *p* < 0.01. “****” indicates *p* < 0.0001.

Neutrophils and monocytes are the main effector cells of the innate immune system. During infection, neutrophils eliminate pathogens mainly through phagocytosis and the release of ROS, but their ability to release inflammatory factors is weaker than monocytes. Here we further explored the effect of age on the intracellular TNF-α secretion capacity of neutrophils and monocytes (Figures 1E,F). The positive proportion of monocytes and neutrophils capable of secreting TNF-α was significantly lower in healthy volunteers compared with patients with abdominal sepsis (*p* < 0.05) (Figure 5). Concerning age, the proportion of neutrophils able to secrete TNF was significantly higher in elderly patients with abdominal sepsis than in nonelderly patients (Figure 5A). However, previous clinical routine blood data showed that the percentage of neutrophils in peripheral blood was significantly higher than that of monocytes, and the percentage of neutrophils in elderly patients was higher, (*p* < 0.05) (Table 1). The percentage of monocytes in the non-elderly group was significantly higher than that in the elderly group.

### Clinical outcomes associated with different age groups

As shown in Table 3, the proportion of patients needing mechanical ventilation was higher in the elderly group (71.4%) than that in the non-elderly group (50.0%). The incidence of acute kidney injury (AKI) was higher in the elderly group (50.0%) than that in the non-elderly group (33.3%) and the length of the hospital stay was 40 (19–44) days in the elderly group and 25 (Esper et al., 2006; Mees et al., 2008; Turnbull et al., 2009; Alves-Filho et al., 2010; Gentile et al., 2012; Prakash et al., 2012; Sadaka et al., 2013; Shaw et al., 2013; Chen et al., 2014; Nacionales et al., 2014; Vanzant et al., 2014; Nacionales et al., 2015; Shalova et al., 2015; Singer et al., 2016; Mira et al., 2017; Kumar, 2018; Raymond et al., 2018; Wang et al., 2018; Coakley et al., 2019; Guo et al., 2019; Peerapornratana et al., 2019; Bartsch et al., 2020; Gameiro et al., 2020; Levey et al., 2020; Mae et al., 2020; Shao et al., 2020; Chen et al., 2021; Yu et al., 2021) days in the mostly non-elderly group. Additionally, the 90-day mortality rate was higher in the elderly group (28.6%) than that in the non-elderly group (16.7%), but no significant differences in these outcomes were observed (*p* > 0.05). We also found that patients in the elderly group spent more time in the ICU [30 (11–41) days] than those in the non-elderly group [21 (12–42) days] (*p* < 0.05).

**TABLE 3:** Outcome measures in patients with abdominal sepsis in different age groups.

Outcome indicator	Total (*n* = 32)	Group A (≥65; *n* = 14)	Group B (<65; *n* = 18)	*p*
MV(%)	19 (59.4)	10 (71.4)	9 (50.0)	0.221
AKI(%)	13 (40.6)	7 (50.0)	6 (33.3)	0.341
RRT (%)	10 (31.3)	6 (42.9)	4 (22.2)	0.212
Vasoactive agent (%)	17 (53.1)	10 (71.4)	7 (38.9)	0.087
Superinfection (%)	8 (25.0)	4 (28.6)	4 (22.2)	0.681
Length of stay (Day)	32 (16–42)	40 (19–44)	25 (11–38)	0.224
Days in the ICU (Day)	13 (8–36)	30 (11–41)	21 (12–42)	0.045
90-day mortality rate(%)	7 (21.9)	4 (28.6)	3 (16.7)	0.419

Note: Group A indicates the cohort of patients aged ≥65 years. Group B indicates the cohort of patients aged <65 years. MV, mechanical ventilation; AKI, acute kidney injury; RRT, renal replacement therapy.

## Discussion

Poor physiological reserve, immune aging, pre-existing conditions, and previous antibiotic exposure in the elderly population all increase the risk of infection, and sepsis is common in the global elderly population. These risk factors partly reflect the increased morbidity and mortality due to infection and non-infectious inflammation seen in the elderly population (Shaw et al., 2013). Sepsis is a life-threatening imbalance of host responses to infection (Singer et al., 2016). Abdominal infection is a common occurrence following surgery and can lead to sepsis (Mazuski et al., 2017). Due to the increasing number of elderly people, it is important to study the impact of age on abdominal sepsis patient outcomes, especially in the context of rising socioeconomic and medical burdens (Bartsch et al., 2020). It is well-known that elderly patients have higher morbidity from abdominal sepsis than younger patients (Chen et al., 2021). In recent years, the mortality rate of abdominal sepsis has decreased with improvements in diagnosis and treatment technology, but the absolute number of deaths is still high because the number of clinical cases increasing, especially those elderly patients who eventually die of complications, including secondary superinfection, multiple organ dysfunction syndrome, septic shock, persistent inflammatory immunosuppression, and catabolic syndrome (Gentile et al., 2012; Vanzant et al., 2014).

In this study, the proportion of elderly patients with underlying diseases was higher than that of non-elderly patients, although the difference between the two was not statistically significant, which may be related to the relatively small sample size of this study. Common underlying diseases in the elderly population include coronary heart disease, congestive heart failure, chronic kidney disease, diabetes, chronic liver failure, chronic obstructive pulmonary disease, malignancies, and chronic infections (Esper et al., 2006). These complications can also mask and delay the diagnosis of sepsis and increase the risk of secondary infection. NLR is an inexpensive and readily available tool that has been shown to provide information on nonspecific systemic inflammation. It has been used to assess malignant tumors (Guo et al., 2019), sepsis (Peerapornratana et al., 2019), kidney diseases (Mae et al., 2020), and even Corona Virus disease 2019 (COVID-19) (Yu et al., 2021). Addition of neutrophil to lymphocyte and platelet ratio (NLPR) to NLR can further improve its clinical value (Gameiro et al., 2020; Levey et al., 2020). In this study, we analyzed clinical data from abdominal sepsis patients by age grouping to further explore the changes of NLR and NLPR. We found that NLRs and NLPRs were both significantly increased in elderly patients, especially NLRs, suggesting that NLRs may be more clinically valuable for elderly abdominal sepsis patients.

The association between NLPR and abdominal sepsis underscores the role of neutrophils, lymphocytes, and platelets in the development of sepsis. We found that the proportion of neutrophils in the elderly group was higher than that of the non-elderly group. A large number of activated neutrophils cannot normally migrate to the site of infection to play the role of eliminating pathogenic microorganisms, but can be transferred to various organs and systems of the body through blood circulation, resulting in corresponding tissue damage (Raymond et al., 2018; Shao et al., 2020). As the main part of adaptive immunity, lymphocytes play an important role in cellular and humoral immunity. Previous studies have found that in the pathogenesis of sepsis, T cell activation continues to fail, and the occurrence of sepsis is closely related to the failure of CD8^+^ helper T cell (Th)-1 and CD4^+^ Th17 lymphocytes (Coakley et al., 2019). At the same time, the up-regulated number of immunosuppressive regulatory T cells (Tregs) accelerates the apoptosis of lymphocytes (Kumar, 2018). Our study found that the lymphocyte count and proportion of elderly patients were lower than those of non-elderly patients, that is, elderly patients with abdominal sepsis were immunosuppressed. Platelets are important mediators that are abundant in the circulatory system and can rapidly release a variety of bioactive substances to regulate inflammation and hemostasis (Gameiro et al., 2020). Red blood cell distribution width (RDW) refers to the coefficient of variation of red blood cell volume. It has been suggested that an increase in admission RDW may be a predictor of short-term mortality in sepsis (Sadaka et al., 2013). In this study, elderly patients had significantly higher elevated RDWs and mortality rates than those in the non-elderly group. In the above studies on RDW and sepsis, the subjects were all adult sepsis patients (including elderly patients, aged ≥65 years), so some scholars considered the effect of age on the results of the study and studied the association between RDW and sepsis patients >65. By studying the clinical data of elderly patients with severe sepsis and (or) septic shock, Wang et al. (2018) found that admission RDW was an independent predictor of in-hospital mortality in septic elderly patients.

Sepsis is currently recognized as causing widespread activation and dysfunction of the innate immune system (Mira et al., 2017). Neutrophils are an important part of the innate immune system of the human body and play an important role in resisting pathogen invasion and immune surveillance of infection. After the onset of sepsis, neutrophils accumulate in numerous sites of sepsis (Alves-Filho et al., 2010), which is beneficial in the early stages of sepsis and can kill pathogens. However, neutrophil **regulation** is complex in abdominal sepsis, and failure of neutrophil migration and shortened lifespan in severe sepsis have been well-established (Prakash et al., 2012). Therefore, as an important component of innate immunity, impaired neutrophil recruitment and migration are related to the pathogenesis and poor prognosis of sepsis. Monocytes play a role in both innate and adaptive immunity. When patients with sepsis are in a state of immunosuppression, the secretion of pro-inflammatory factors by monocytes decreases, while the secretion of anti-inflammatory factors increases, resulting in the phenomenon of “endotoxin tolerance” in which endotoxin phagocytosis is weakened (Shalova et al., 2015). Monocytes can also participate in adaptive immunity by acting as antigen-presenting cells to initiate T cell-specific differentiation.

Multiple aspects of innate immunity have been recently identified to be essential for the survival of sepsis, and this response is not clear in the elderly. In this study, we also found that the proportion of neutrophils and monocytes increased in patients with sepsis. We found that neutrophils increased more significantly in elderly patients and monocytes increased more significantly in non-elderly patients, but the proportion of neutrophils was significantly higher than monocytes. We observed that circulating phagocytes exhibit increased phagocytic function and bactericidal capacity, however neutrophils show decreased resting production of ROS while monocytes have increased oxidative stress. It was found that there was no difference in the level of lung injury between juvenile and aged mice after *P. pneumoniae* infection in a septic mouse model (Nacionales et al., 2015). However, bronchoalveolar lavage fluid samples after sepsis showed defective neutrophil function in aged animals. These defects were not found to include a decrease in ROS production though there was a significant decrease in leukocytes in the lungs after sepsis and pneumonia, indicating a defect in phagocyte recruitment in the elderly compared to the young (Chen et al., 2014). Phagocytosis analysis indicated that less phagocytic activity but more cells doing phagocytosis (though weak). In this study, monocytes in elderly septic patients have markedly increased release of ROS, providing evidence for an overwhelming nonspecific hyperinflammatory state in the early phase of sepsis, which leads to hypoperfusion and tissue damage in the early phase of sepsis. This further explains the reason for elderly patients requiring higher organ support conditions and with lower survival rates in abdominal sepsis. Clearly, patients with severe infections who develop multiple organ failure often suffer from immune dysfunction (Nacionales et al., 2014). It has been hypothesized that higher age exacerbates these immune impairments (Mees et al., 2008), but the mechanism remains unclear. After intraperitoneal sepsis, the cytokine response in aged mice was similar to that in young mice (Turnbull et al., 2009). This was also confirmed by our results, where we found that cytokines secreted by both neutrophils and monocytes increased after abdominal sepsis; after grouping by age, the ability of neutrophils to secrete cytokines was significantly enhanced in the elderly group, with little difference in monocytes between the elderly and non-elderly groups, but the proportion of neutrophils in whole blood cells was significantly higher than that in monocytes. It has been shown that during infection phagocyte function does not decline in all patients, especially during the development of organ failure, with increased production of proinflammatory cytokines (Taylor et al., 2014).

## Conclusion

Patients classified as elderly are more likely to have underlying organ dysfunction, low immunity, etc., putting them at greater risk for developing sepsis and having long-term health deficits. In this context, our study focused on the effect of aging on the clinical sepsis characteristics and immune function. We found that elderly patients have decreased immune cell function and increased release of cytokines compared to younger patients, suggesting individualized treatment plans targeting the elderly septic microenvironment could help prevent organ failure in elderly septic patients and improve patient survival.

## Data Availability

The original contributions presented in the study are included in the article/Supplementary Material, further inquiries can be directed to the corresponding author.
